# L-arginine alters myogenic genes expression but does not affect breast muscle characteristics by in ovo feeding technique in slow-growing chickens

**DOI:** 10.3389/fvets.2022.1030873

**Published:** 2022-12-14

**Authors:** Panpan Lu, Thanidtha Morawong, Amonrat Molee, Wittawat Molee

**Affiliations:** School of Animal Technology and Innovation, Institute of Agricultural Technology, Suranaree University of Technology, Nakhon Ratchasima, Thailand

**Keywords:** L-arginine, in ovo feeding, muscle development, gene expression, slow-growing chicken

## Abstract

In ovo feeding (IOF) of nutrients is a viable method for increasing muscle mass through hyperplasia and hypertrophy. The objective of this study was to evaluate the effects of IOF of L-arginine (Arg) on breast muscle weight, muscle morphology, amino acid profile, and gene expression of muscle development in slow-growing chickens. Four hundred eighty fertilized eggs were randomly divided into two groups: the first group was the non-injected control group, and the second group was the Arg group, injected with 1% Arg (0.5 mL) into the amnion on day 18 of incubation. After hatching, 160 birds from each group were randomly divided into four replicates of 40 birds each. This experiment lasted for 63 days. The results showed that IOF of Arg did not affect (*P* > 0.05) breast muscle weight, muscle morphology, and mRNA expression of mammalian target of rapamycin (*mTOR*) signaling pathway in slow-growing chickens. However, the amino acid profile of breast muscle was altered (*P* < 0.05) on the day of hatching (DOH), day 21 (D21), and day 42 (D42) post-hatch, respectively. Myogenic factor 5 (*Myf5*) mRNA expression was upregulated (*P* < 0.05) on D21 post-hatch. Myogenic regulator 4 (*MRF4*) mRNA expression was increased (*P* < 0.05) on DOH. And myogenin (*MyoG*) was increased (*P* < 0.05) on DOH and D21 post-hatch, in the Arg group compared to the control group. Overall, IOF of 1% Arg improved the expression of myogenic genes but did not influence muscle morphology and BMW. These results indicate that in ovo Arg dosage (0.5 mL/egg) has no adverse effect on breast muscle development of slow-growing chickens.

## Introduction

In recent years, the meat of slow-growing chickens has gained popularity and is recognized globally for its excellent meat quality (1, 2). However, they produce less meat (especially breast meat) compared to fast-growing chickens (3). This disadvantage limits their development in the future. To promote poultry productivity and improve market competitiveness, this study needs to develop a strategy to increase the meat yield of slow-growing chickens.

Meat yield is an important economic trait that attributes to the increase of skeletal muscle mass, and which depends on the increase of the number (hyperplasia) and size (hypertrophy) of muscle fibers. In birds, the number of muscle fibers is fixed at hatching. After hatching, muscle development occurs by increasing of muscle fiber size. The skeletal muscle fibers are derived from satellite cells (also called myoblasts) and their proliferative progeny can be marked by paired box 7 (*Pax7*) (4). Then, these satellite cells proliferate, differentiate, and fuse into myotubes and then incorporate into existing adjacent fibers, resulting in muscle fiber hypertrophy (5). This process is regulated by myogenic regulatory factors (MRFs) that specify muscle fiber development, including myogenic differentiation factor 1 (*MyoD*), myogenic factor 5 (*Myf5*), myogenic regulator 4 (*MRF4*), and myogenin (*MyoG*) (6). Mammalian target of rapamycin (mTOR) is a vital signaling pathway to mediate protein synthesis for cell proliferation and muscle hypertrophy by activating its downstream targets of ribosomal protein S6 kinase 1 (*S6K1*) and eIF4E-binding protein 1 (*4EBP1*) (7). Obviously, there is a close relationship among these genes for muscle fiber hypertrophy and muscle mass (8, 9). In consideration of the above characters of muscle development. It is suggested that the addition of exogenous nutrients to the embryo may be a novel way to increase muscle mass through hyperplasia and hypertrophy of muscle fibers.

In ovo feeding (IOF) is a viable method that delivers appropriate nutrients to the amnion at the later stages of the embryo (10, 11). Numerous studies have demonstrated that IOF of exogenous nutrients plays a positive role in embryonic development and post-hatching performance in poultry (12–15). L-arginine (Arg) is a nutritionally essential amino acid for poultry and has many biological and physiological functions (16), one of the primary functions is to synthesize protein for supporting cell growth. Previous studies have shown that IOF of Arg can activate the mTOR signaling pathway and protein accumulation in broilers in the starter period (17–19). *In vitro* experiments indicated that Arg stimulates myogenesis in broiler muscle (20). Furthermore, Arg has a positive effect on muscle growth in poultry (21–23). However, studies on IOF of nutrients in slow-growing chickens are rare.

The Korat chicken (KRC) is one of the slow-growing chicken breeds. It was crossbred from the indigenous line (Leung Hang Khao) and the Suranaree University of Technology (SUT) line. The meat of KRC has high collagen and good texture (24), and the body weight can get to around 1.2 kg at 9 weeks of age. KRC is a representative of the slow-growing chickens in Thailand and is supported by a national policy that encourages farmers to increase their incomes. To our knowledge, we hypothesize that IOF of Arg can improve breast muscle weight (BMW) by hyperplasia and hypertrophy of muscle fiber at the market age. Moreover, it is unclear how IOF of Arg regulates the gene expression patterns of muscle development for muscle fiber hypertrophy post-hatch in slow-growing chickens.

Therefore, the present study aimed to evaluate the effects of IOF of Arg on BMW, muscle morphology, amino acid profile, and gene expression of muscle development in slow-growing chickens.

## Materials and methods

All experimental protocols were approved by the Ethics Committee on Animal Use of the SUT, Nakhon Ratchasima, Thailand (user application ID: U1-02633-2559).

### Eggs and incubation

Four hundred eighty fertilized KRC eggs (Leung Hang Khao males and SUT females) were obtained from the SUT farm (Nakhon Ratchasima, Thailand). All eggs (range = 54–60 g) were randomly distributed in an incubator (Model Pet. 192-IV; Petersime Incubation Equipment Co., Ltd., Zulte, Belgium). The optimal incubation condition was maintained at 37.8°C with 60% relative humidity, and eggs were turned automatically per hour.

### Preparation of the solution and IOF procedure

One percent Arg solution was freshly prepared according to a previous study (25) with modification; that is, 1.5 g Arg (Sigma-Aldrich Inc., St. Louis, MO, USA) was dissolved in 150 mL of 0.9% saline, it equivalent to 5 mg of Arg per egg. The Arg solution was sterilized by autoclaving at 120°C for 15 min and then nullified through a 0.45 μm membrane filter.

On day 18 of incubation, all eggs were candled by an electric torch, and unfertilized eggs were removed and discarded. Meanwhile, 480 embryonated eggs (59.0 ± 1.0 g) was randomly divided into two groups. The first group was the non-injected group (control group), and the second group was injected with 1% Arg (Arg group). The large end surface of eggs in the Arg group were disinfected with 75% alcohol. The pinhole was punched and 0.5 mL of Arg solution (5 mg/egg) was injected into the amnion using a 21-gauge needle (26). The eggs of the control group were kept outside the incubator with the same environmental conditions as those of the Arg group. After injection, the holes were sealed with paraffin wax. The eggs of each treatment were randomly divided into four replicates with 60 eggs each, and each basket was a replicate. The eggs were placed in the hatching baskets and transferred to the hatcher to perform the hatchery program.

### Bird rearing

After hatching, a total of 160 mixed-sex and healthy birds from each group with similar body weight (BW) close to the mean BW of the pooled group were randomly divided into four replicates of 40 birds each. Each treatment group was replicated in 4 pens, total eight litter floor pens were provided in this experiment, and each pen was regarded as a replicate. Each replicate was reared separately in an open-sided house with natural ventilation. The experiment lasted for 63 days. All birds were provided ad libitum the commercial corn-soybean meal feed (Charoen Pokphand Co., Ltd., Nakhon Ratchasima, Thailand) and fresh water. The nutrient contents of feed for the three feeding periods were determined by the AOAC methods (27) and are shown in Table 1. The vaccination program for the birds was carried out according to SUT farm guidelines.

**Table 1:** Analyzed nutrient composition of the basal diets (as-fed basis, % unless stated otherwise).

**Items**	**Starter diet (DOH–D21)**	**Grower diet (D22–D42)**	**Finisher diet (D43–D63)**
Dry matter	93.83	93.51	94.21
Gross energy, (MJ/Kg)	12.54	12.96	13.38
Crude protein	22.72	20.46	18.65
Crude fat	5.20	6.74	6.66
Crude fiber	3.44	3.45	3.55
Ash	4.70	4.58	4.19
Lysine	1.78	1.43	0.92
Methionine	0.34	0.25	0.28
Threonine	1.01	0.85	0.73
Arginine	1.58	1.13	0.55

### Tissue collection

On the day of hatching (DOH), day (D21), day (D42), and day (D63) post-hatch, eight birds per group (two male birds per replicate) with similar mean BW of their pen were selected, weighed, and killed after using chloroform. The entire breast muscle was weighed and small parts of the breast muscle were fixed in 4% paraformaldehyde for morphological analysis. The pectoralis major of the breast muscle was collected, frozen in liquid nitrogen, and stored at −80°C for amino acid and mRNA expression analyses.

### Morphological observation

The breast muscle samples were fixed in 10% buffered formalin at room temperature for 24 h, and were dehydrated by different concentration of ethanol, and cleared in xylene, then a small cube was embedded in paraffin, the sections of 5 μm thickness were cut by a cryostat and mounted on glass slides, the sections were dewaxed with xylene, and hydrated by ethanol. Then the samples were stained with hematoxylin and eosin. All sections of the breast muscle were observed and five representative sections per sample were monitored under a light microscope (Olympus CX21, United States). The muscle fiber number (MFN) and muscle fiber diameter (MFD) were measured in surface of 1 mm^2^ cross-section of the sample using an image analyzer (Image-Pro Plus 5.0 software, Media Cybernetics Inc., Bethesda, MD, USA).

### Amino acid analysis

Amino acid content was measured as previously described (28) with modifications. Fresh breast muscles (80 mg) were placed in 10 mL hydrolysis tubes with 4 mL of 6 M hydrochloric acid solution, filled with nitrogen gas for 5 min, and then transferred to the heating incubator at 110°C for 24 h. The supernatant was transferred to centrifuge tubes through 0.45 μm membrane filters for ready use. Then 1 μL of the solution was prepared for injection, including 40 μL internal standard (norleucine), 250 μL sample supernatant, and 710 μL buffer, and measured using an ultra-ninhydrin solution at detection wavelength of 570 nm by an amino acid analyzer (Biochrom 30+, Cambridge, UK). The amino acid content was expressed as milligrams per gram of breast muscle.

### Total RNA isolation and reverse transcription

Total RNA was isolated from breast muscle using TRIzol Reagent (Invitrogen, Thermo Fisher Scientific). The purity and concentration of RNA were measured using a NanoDrop 2,000 spectrophotometer (Thermo Fisher Scientific). The ratio of OD 260/280 was between 1.8 and 2.0, which was considered qualified. The RNA integrity was verified using polyacrylamide gel electrophoresis. DNase I was used to eliminate DNA contamination. Complementary DNA was synthesized in 20 μL volume with 1 μg RNA using a reverse transcription Kit (Applied Biosystems; Thermo Fisher Scientific) according to the manufacturer's instructions.

### Real-time PCR

The primers for the target genes were designed using Primer Premier 5 software according to the mRNA sequences of *Gallus gallus* in the NCBI database and synthesized by Gibthai (Bangkok, Thailand). The endogenous reference gene was glyceraldehyde 3-phosphate dehydrogenase (GAPDH) based on the recommendation of a previous study (29). The sequences are presented in Table 2. The mRNA expression of these genes was quantified by real-time PCR using a Light Cycler 480 System (Roche Diagnostics GmbH, Mannheim, Germany). This reaction system was performed in a 20 μL volume, containing 10 μL of SYBR Green Master Mix (Applied Biosystems, Thermo Fisher Scientific), 2 μL of cDNA (diluted 1:10), 1 μL of forwarding primer (10 μM), 1 μL of reverse primer (10 μM) and 6 μL of diethylpyrocarbonate-treated water. The cycling conditions for real-time PCR were as follows: initial denaturation at 95°C for 10 min, 40 cycles of denaturation at 95°C for 30 s, the annealing temperature of specific primers for 35 s, and final dissociation stages at 95°C for 5 s and 72°C for 5 min, and the melting curves were analyzed to determine the specificity of all target genes. The experiment for each sample of target genes was carried out in triplicate. The difference in mRNA expression of each target gene was calculated using the 2^−ΔΔct^ method (30), and the expression of the *GAPDH* gene was used as an internal control.

**Table 2:** Primer sequences used for real-time PCR.

**Gene**	**Gene bank** **accession no**.	**Primers sequence (5^′^-3^′^)**	**Product size (bp)**
*mTOR*	XM_417614.6	F:CTCAGCGGGAACCAAAAGA	125
		R:ATGGATTCGGTCATCACGG	
*S6K1*	NM_001030721.1	F:GAGGAGTGGGCATAATCGTG	154
		R:TGTGAGGTAGGGAGGCAAAT	
*4EBP1*	XM_424384.6	F:CCTGATGGAGTGCCGTAAT	77
		R:GGCTGGTAACACCTGGAAT	
*Pax7*	NM_205065.1	F:CAAAGGGAACAGGCTGGATG	102
		R:TGCTCGGCAGTGAAAGTGGT	
*MyoD*	L34006.1	F:CACAGTCACCGTTTTCCCA	102
		R:GCCTCACAGCACAAGCATC'	
*Myf5*	NM_001030363.1	F:GAGGAGGAGGCTGAAGAAAG	119
		R:CGATGTACCTGATGGCGTT	
*MyoG*	NM_204184.1	F:GGATGGTGATGCTGGAAGGA	93
		R:GGAAAGGATTTGGGCGGTT	
*MRF4*	D10599.1	F:AGGCTCTGAAAAGGCGGACT	169
		R:TTGGGGCTGAAGCTGAAGG	
*GAPDH*	NM 204305.1	F:GAGGGTAGTGAAGGCTGCTG	113
		R:CATCAAAGGTGGAGGAATGG	

mTOR, mammalian target of rapamycin; S6K1, ribosomal protein S6 kinase 1; 4EBP1, eIF4E-binding protein 1; Pax7, paired box 7; MyoD, myogenic differentiation 1; Myf5, myogenic factor 5; MyoG, myogenin; MRF4, myogenic regulator 4; GAPDH, glyceraldehyde 3-phosphate dehydrogenase.

### Statistical analysis

The data were analyzed by independent *t*-tests using SPSS software (version 22.0, IBM Corp. 1989, 2013, New York, USA), and the statistical significances between the two groups were denoted as *P* < 0.05. Additionally, principal component analysis (PCA) was performed to classify the samples from the two groups for visualizing the underlying data structure by using the Unscrambler X 10.5 software (CAMO Software, Oslo, Norway). Heatmaps of Pearson correlation were conducted to assess the relationships between the breast muscle characteristics (BMW and MFD) and gene expression of muscle development using Graph-Pad Prism version 8.0 (GraphPad Software Inc., San Diego, CA).

## Results

### Breast muscle weight and muscle morphology

In Table 3, the IOF of Arg did not affect (*P* > 0.05) BMW and muscle morphology (MFN and MFD) from DOH to D63 post-hatch, compared to the control group.

**Table 3:** In ovo feeding of L-arginine on breast muscle weight and muscle morphology in slow-growing chickens.

**Items**	**Treatments**		**SEM**	***P*-value**
	**Control**	**Arg**		
**Breast muscle weight, (%)**				
DOH	1.20	1.21	0.016	0.788
D21	7.05	7.31	0.222	0.427
D42	8.59	8.69	0.180	0.694
D63	10.90	11.74	0.281	0.148
**Muscle fiber diameter, (μm)**				
DOH	5.69	5.95	0.138	0.272
D21	18.03	20.11	0.832	0.199
D42	22.78	21.56	0.847	0.429
D63	34.10	34.43	0.355	0.599
Muscle fiber number, D63, (per mm^2^)	465.02	438.26	0.134	0.253

Breast muscle weight (%) = the ratio of the breast muscle weight to BW.

DOH, day of hatching; D21, day 21; D42, day 42; D63, day 63.

Control, non-injected group; Arg, 1% L-arginine-injected group.

SEM, standard error of the mean. Values are means with n = 8 per treatment.

### Amino acid profile

**Figure 1:**
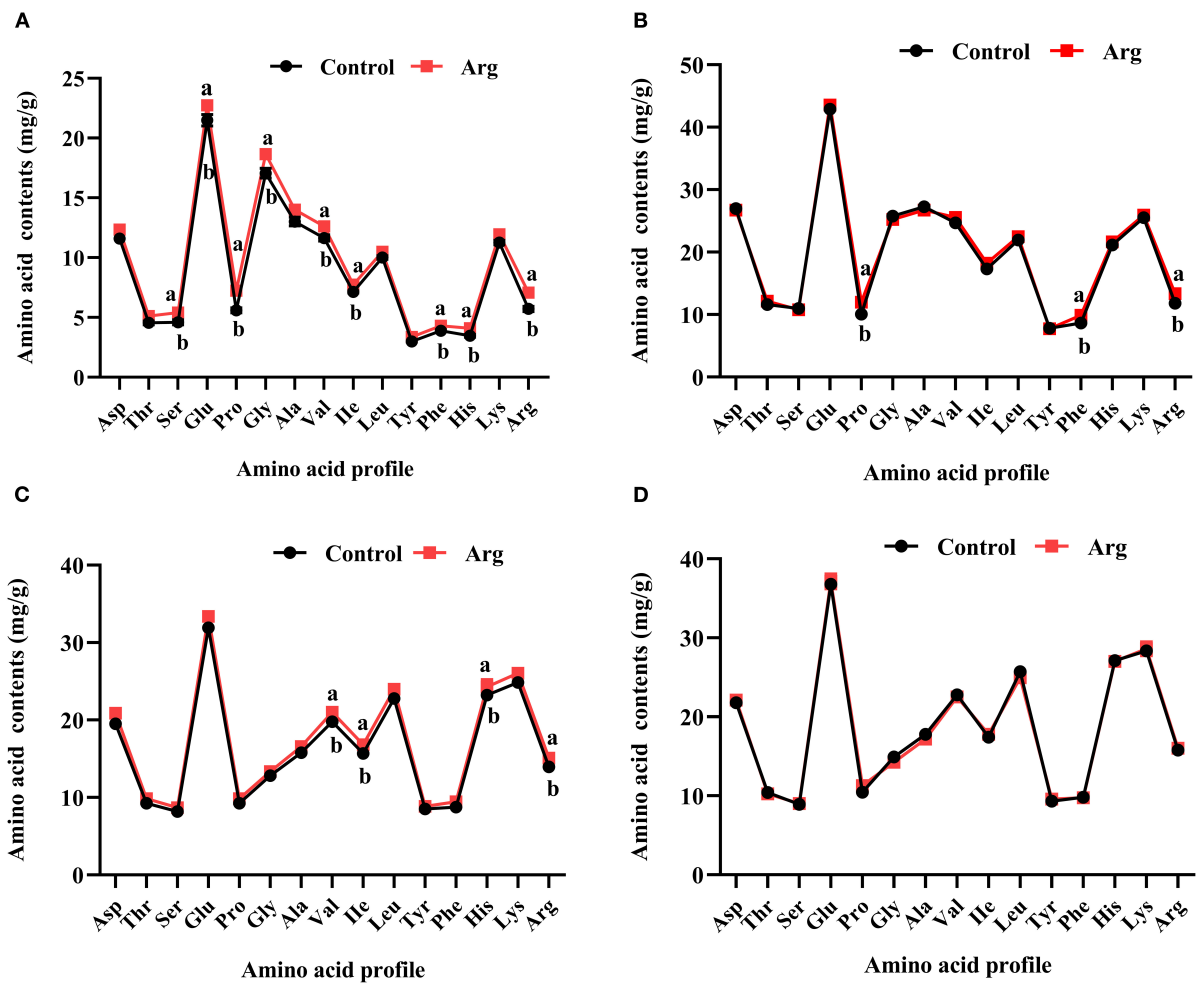
Effects of in ovo feeding of L-arginine on the changes of amino acid profile in the breast muscle of slow-growing chickens. **(A)** Day of hatching; **(B)** day 21; **(C)** day 42; **(D)** day 63. Control, non-injected group; Arg, 1% L-arginine-injected group. Asp, aspartic acid; Thr, threonine; Ser, serine; Glu, glutamic acid; Pro, proline; Gly, glycine; Ala, alanine; Val, valine; Ile, isoleucine; Leu, leucine; Tyr, tyrosine; Phe, phenylalanine; His, histidine; Lys, lysine; Arg, arginine. SEM, standard error of the mean. Values are means with *n* = 8 per treatment. Different superscripts with the same time point indicate significant differences between the two groups (*P* < 0.05).

As shown in Figure 1A, the amino acid contents (serine, glutamic acid, proline, glycine, valine, isoleucine, phenylalanine, histidine, and arginine) in the breast muscle on DOH were significantly higher (*P* < 0.05) owing to the IOF of Arg. The contents of proline, phenylalanine, and arginine were significantly higher (*P* < 0.05) in the breast muscle on D21 post-hatch owing to IOF of Arg (Figure 1B). Moreover, the IOF of Arg significantly increased (*P* < 0.05) the contents of valine, isoleucine, histidine, and arginine in the breast muscle on D42 (Figure 1C) post-hatch and had no effect (*P* > 0.05) on D63 post-hatch (Figure 1D), compared to the control group.

### Gene MRNA expression related to the muscle development

**Figure 2:**
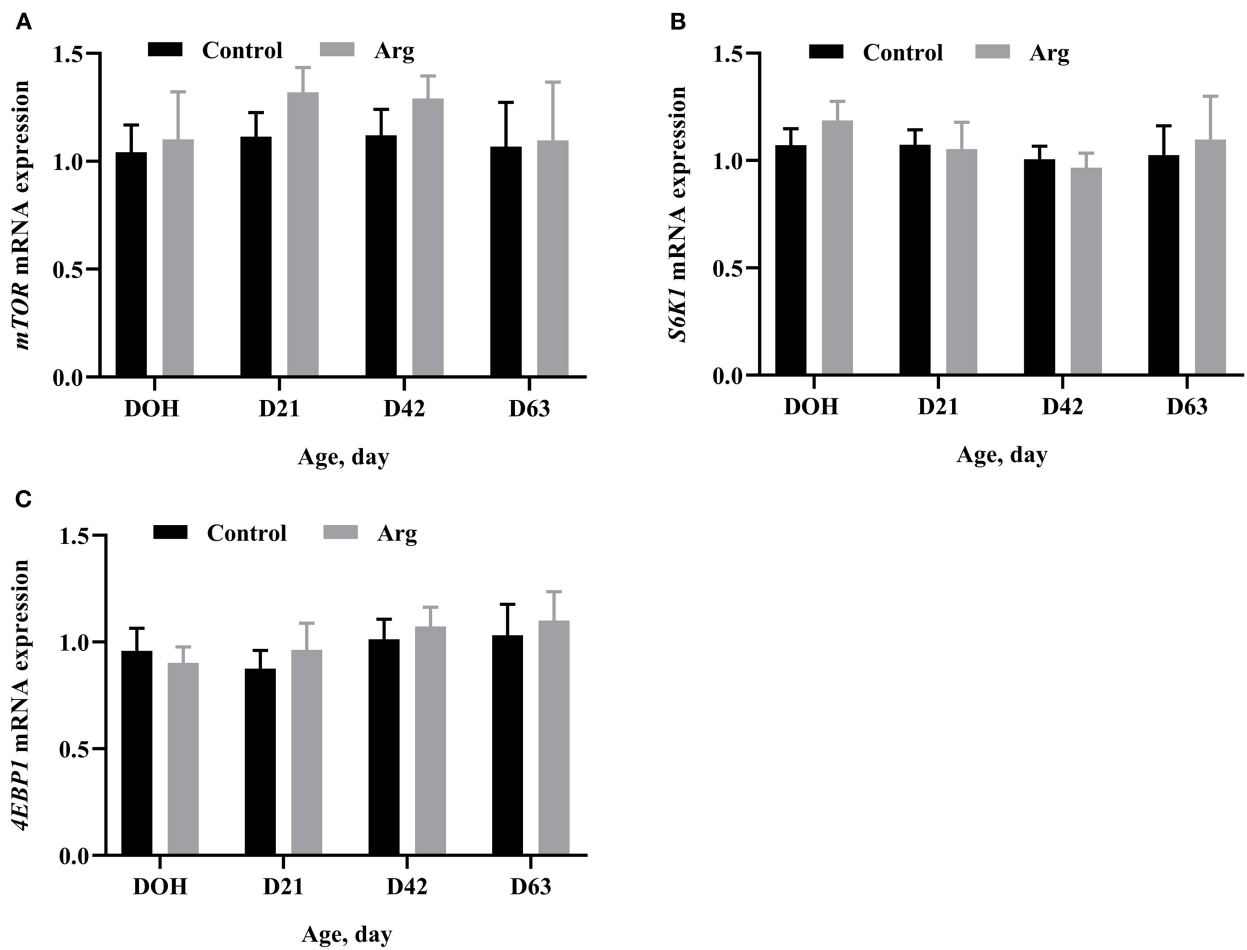
Effects of in ovo feeding of L-arginine on gene mRNA expression related to the mTOR signaling pathway on DOH, D21, D42, and D63 post-hatch in slow-growing chickens. **(A)** *mTOR*, mammalian target of rapamycin; **(B)** *S6K1*, ribosomal protein S6 kinase 1; **(C)** *4EBP1*, eIF4E-binding protein 1. DOH, day of hatching; D21, day 21; D42, day 42; D63, day 63. Control, non-injected group; Arg, 1% L-arginine-injected group. SEM, standard error of the mean. Values are means with *n* = 8 per treatment. Different superscripts indicate significant differences between the two groups (*P* < 0.05).

In Figure 2, the mRNA expression of *mTOR, 4EBP1*, and *S6K1* of the breast muscle did not differ (*P* > 0.05) between the Arg and control groups from DOH to D63 post-hatch.

**Figure 3:**
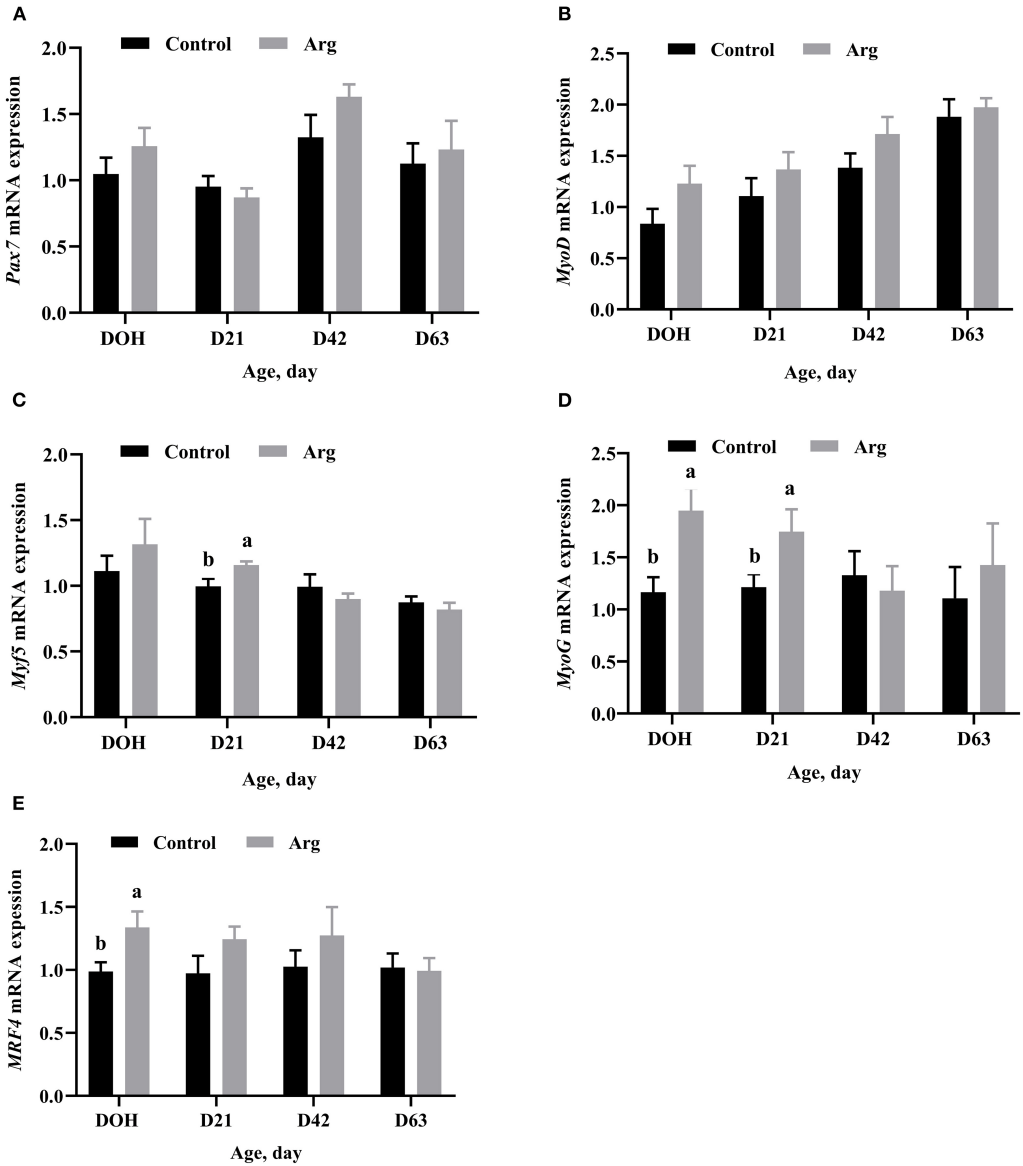
Effects of in ovo feeding of L-arginine on gene mRNA expression related to myogenic regulatory factors on DOH, D21, D42, and D63 post-hatch in slow-growing chickens. **(A)** *Pax7*, paired box 7; **(B)** *MyoD*, myogenic differentiation 1; **(C)** *Myf5*, myogenic factor 5; **(D)** *MyoG*, myogenin; **(E)** *MRF4*, myogenic regulator 4. DOH, day of hatching; D21, day 21; D42, day 42; D63, day 63. Control, non-injected group; Arg, 1% L-arginine-injected group. SEM, standard error of the mean. Values are means with *n* = 8 per treatment. Different superscripts with the same time point indicate significant differences between the two groups (*P* < 0.05).

Gene mRNA expression related to MRFs of the breast muscle is shown in Figure 3. There were no significant differences (*P* > 0.05) in the mRNA expression of *Pax7* and *MyoD* in the breast muscle between the two groups from DOH to D63 post-hatch. The mRNA expression of *Myf5* was significantly upregulated (*P* < 0.05) in the Arg group on D21 post-hatch. The mRNA expression of *MyoG* was higher (*P* < 0.05) in the Arg group on DOH and D21, respectively. The mRNA expression of *MRF4* was higher (*P* < 0.05) in the Arg group on DOH compared to the control group.

### Principal component analysis

**Figure 4:**
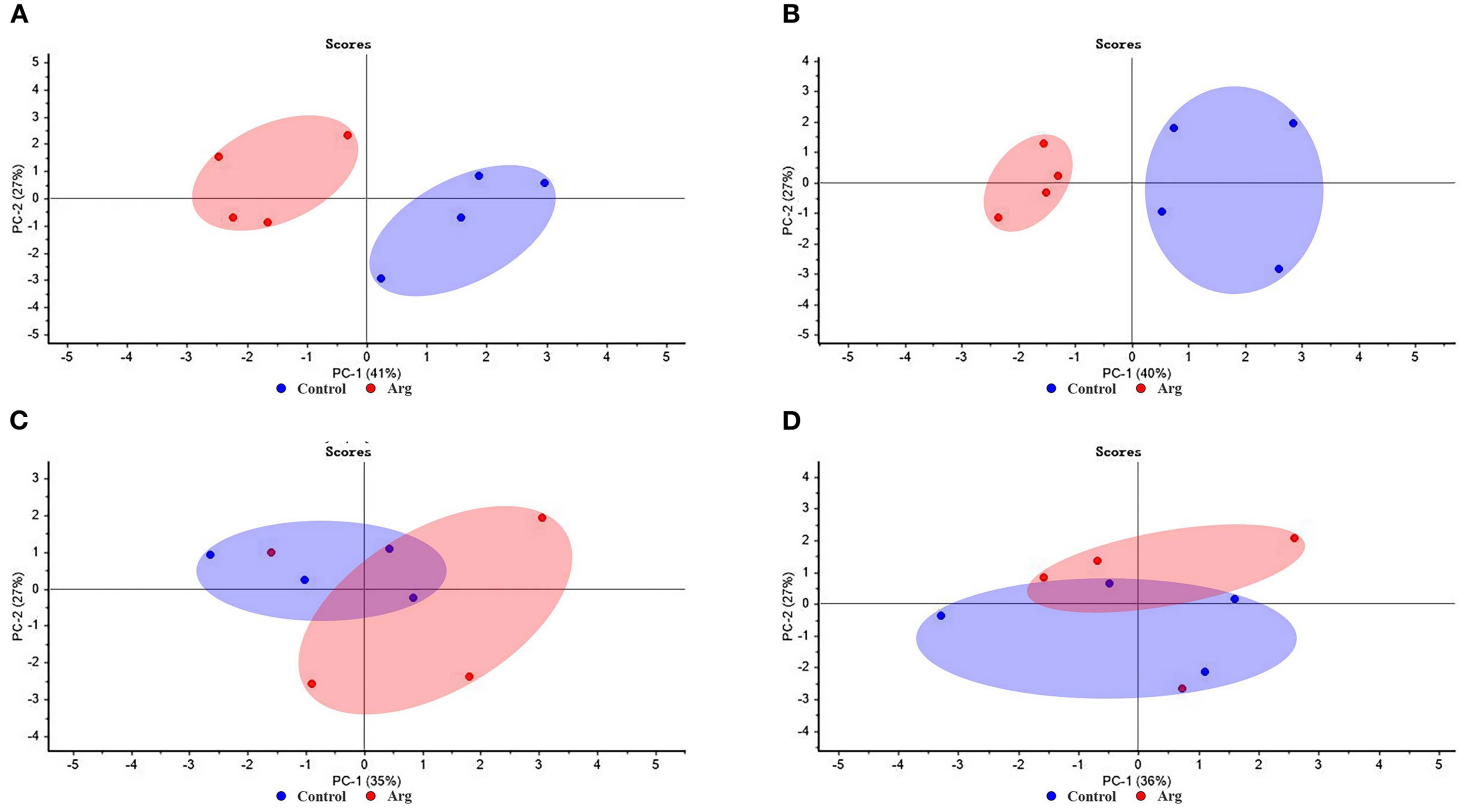
Score plot of principal component analysis (PCA) for breast muscle characteristics and gene expression of muscle development from the two groups in slow-growing chickens. **(A)** Day of hatching; **(B)** day 21; **(C)** day 42; **(D)** day 63. Control, non-injected group; Arg, 1% L-arginine-injected group.

PCA score of breast muscle characteristics (BMW and MFD) and gene expression of muscle development (*Pax7, MyoD, Myf5, MyoG, MRF4, mTOR, 4EBP1*, and *S6k1*) from the two groups in slow-growing chickens are presented in Figure 4. As shown in Figures 4A,B, PC1 explained 41 and 40% of variances, respectively. It separated the clustering of samples from the control and Arg groups on DOH and D21, respectively. As shown in Figures 4C,D, there was no clear separation between the control and Arg groups on D42 and D63, respectively.

### Correlation analysis

**Figure 5:**
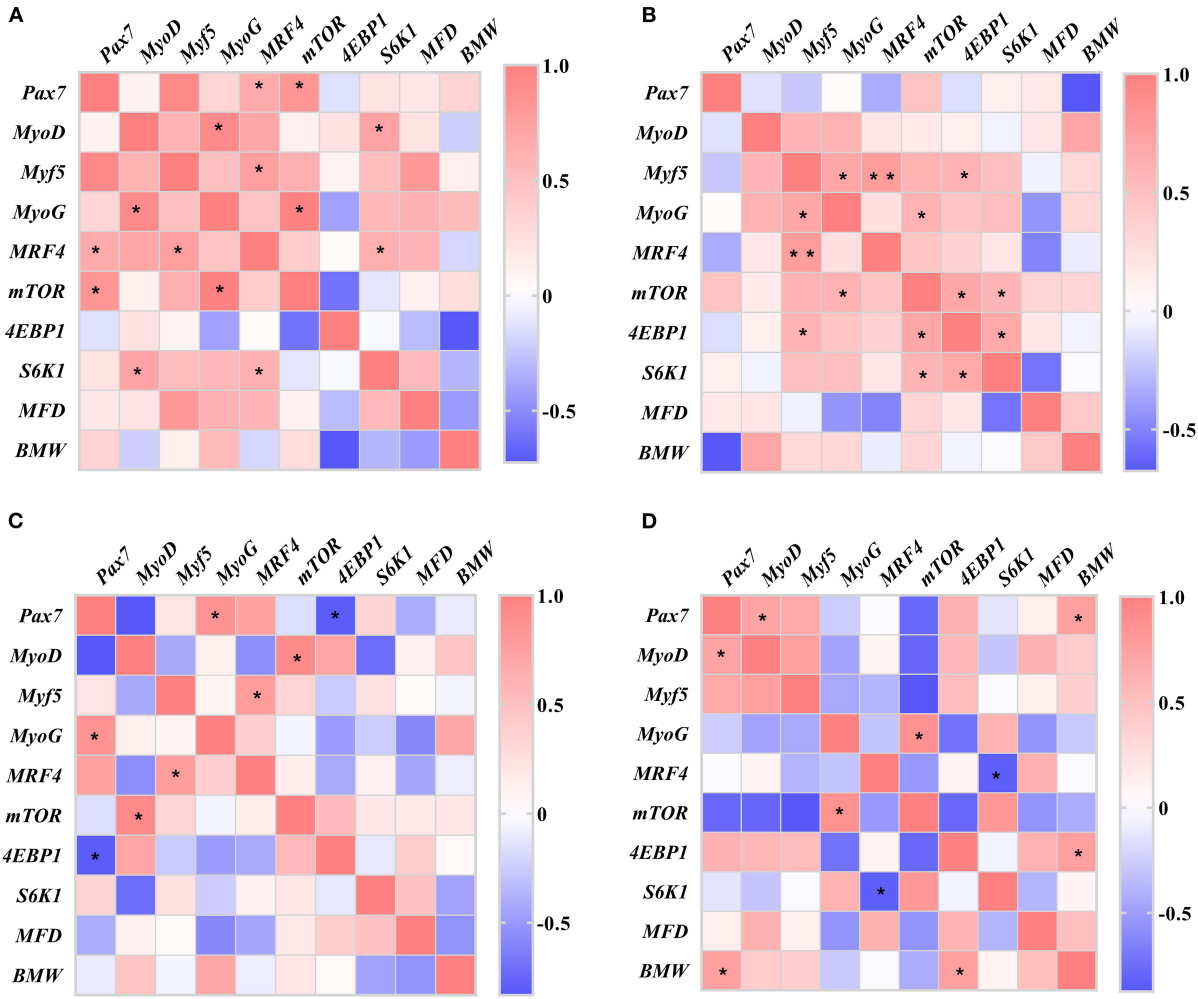
Heatmaps of correlation between the breast muscle characteristics and genes of muscle development in slow-growing chickens. **(A)** Day of hatching; **(B)** day 21; **(C)** day 42; **(D)** day 63. *Pax7*, paired box 7; *MyoD*, myogenic differentiation 1; *Myf5*, myogenic factor 5; *MyoG*, myogenin; *MRF4*, myogenic regulator 4; *mTOR*, mammalian target of rapamycin; *4EBP1*, eIF4E-binding protein 1; *S6K1*, ribosomal protein S6 kinase 1; MFD, muscle fiber diameter; BMW, breast muscle weight. *P < 0.05, **P < 0.01.

The results of the correlation analysis between breast muscle characteristics (BMW and MFD) and gene expression of muscle development (*Pax7, MyoD, Myf5, MyoG, MRF4, mTOR, 4EBP1*, and *S6k1*) are presented in Figure 5.

On DOH, the expression of *MyoG* showed a positive correlation with *MyoD* gene (*r* = 0.909, *P* = 0.032). The expression of *MRF4* gene was positively correlated with *Pax7* (*r* = 0.657, *P* = 0.020) and *Myf5* (*r* = 0.758, *P* = 0.011) genes, respectively. The expression of *mTOR* gene also had positive correlation with *Pax7* (*r* = 0.834, *P* = 0.010) and *MyoG* (*r* = 0.984, *P* = 0.016) genes, respectively. Whereas *S6K1* gene expression was positively correlated with *MyoD* (*r* = 0.758, *P* = 0.018) and *MRF4* (*r* = 0.758, *P* = 0.028) genes, respectively.

On D21, the *Myf5* gene expression was positively correlated with *MyoG* (*r* = 0.690, *P* = 0.027), *MRF4* (*r* = 0.773, *P* = 0.003), and *4EBP1* (*r* = 0.597, *P* = 0.040) genes, respectively. The expression of *MyoG* gene had positive correlation with *mTOR* gene (*r* = 0.605, *P* = 0.037). And the expression of *mTOR* gene was positively correlated with *4EBP1* (*r* = 0.697, *P* = 0.012) and *S6K1* (*r* = 0.583, *P* = 0.047) genes, respectively. A positive correlation was obtained between *4EBP1* and *S6K1* genes expression (*r* = 0.674, *P* = 0.027).

On D42, the expression of *Pax7* gene was positively correlated with *MyoG* gene (*r* = 0.851, *P* = 0.032) and negatively correlated with *4EBP1* gene (*r* = −0.813, *P* = 0.049), respectively. The *MyoD* gene expression had a positive correlation with *mTOR* gene (*r* = 0.915, *P* = 0.029). Whereas *Myf5* gene expression showed a positive correlation with *MRF4* gene (*r* = 0.776, *P* = 0.040).

On D63, the gene expression of *Pax7* was positively correlated with *MyoD* gene (*r* = 0.718, *P* = 0.045) and BMW (*r* = 0.755, *P* = 0.030), respectively. The *MyoG* gene expression also had a positive correlation with *mTOR* gene (*r* = 0.865, *P* = 0.026). A negative correlation was found between *MRF4* and *S6K1* genes (*r* = −0.835, *P* = 0.010). In addition, the *4EBP1* gene expression was positively correlated with BMW (*r* = 0.754, *P* = 0.031).

## Discussion

In the present study, the IOF of Arg had no effect on BMW and muscle morphology (MFN and MFD). These results are in agreement with those of Li et al. (20), who demonstrated that IOF of different levels of Arg had no positive influence on the breast muscle of broiler chickens in the starter period. In contrast, some studies reported that IOF of 1% Arg increased BMW in poultry during D21 post-hatch (18, 22, 31). The differences between these studies could be due to the different genetic backgrounds, Arg concentrations, volumes, and injection times. The increase in MFD is an indicator of the increase in muscle mass by the accumulation of more satellite cells. Castro et al. (23) reported that Arg supplementation in broiler chickens did not improve MFD on D42. Fernandes et al. (21) also indicated that dietary supplementation with Arg responded positively to MFD of broiler chickens in the starter period. Arg is not only involved in protein synthesis, but also regulates energy metabolism by being converted to glucose *via* gluconeogenesis (25, 32). However, further studies are needed to determine whether Arg plays a role in energy metabolism for the energy supply of slow-growing chickens, which could explain the results of the present study.

Changes in amino acid profile respond to variations in muscle mass. Previous reports have shown that Arg can be absorbed and entered the blood and muscles to alter their amino acid profile, then increased BMW and body growth during the starter period post-hatch in broiler chickens by IOF administration (18, 33). The results of the present study are consistent with previous studies and showed that Arg administration to the embryo modulated the amino acid profile in muscle until the grower period post-hatch in slow-growing chickens. Ohta et al. (34) also reported that IOF of the amino acids in the egg increased amino acid accumulation and stimulated amino acid utilization by increasing amino acid synthesis and decreasing amino acid degradation for maximum growth. In addition, Al-Murrani (35) found that supplementing the embryo with amino acids of the same profile as in the egg resulted in increased chick weight until D56 post-hatch. Combined with the results of BMW and muscle morphology, our results imply that changes in amino acid profiles may not improve amino acid utilization by IOF of Arg. This could be due to the amino acid profiles of breast muscle being different from those in the embryo and cannot respond to muscle growth from DOH to D42 post-hatch in slow-growing chickens.

Arg is able to stimulate protein synthesis by the mTOR signaling pathway. However, the results of the present study did not differ by IOF of Arg. This result is not consistent with the previous pieces of literature; some studies have shown that Arg activated the mTOR signaling pathway by in ovo administration in broiler chickens or *in vitro* experiments (17, 18, 36). Earlier studies have also shown that the Arg diet activated the mTOR signaling pathway and inhibited the gene expression of protein degradation in layers (37, 38), due to protein deposition depends on the positive balance between protein synthesis and degradation (39). In addition, Arg can secrete growth hormone (GH), insulin-like growth factor-1 (IGF-1), and insulin (40), which are involved in the mTOR signaling pathway and stimulate protein synthesis (41, 42). Xu et al. (43) revealed that dietary Arg increased the secretion of GH, IGF-1 and insulin and enhanced growth performance in broiler chickens. Arg and GH/IGF-1/insulin might have a joint effect on protein synthesis. We, therefore, speculate that the mTOR signaling pathway in slow-growing chickens is controlled by Arg and GH/IGF-1/insulin. Further studies should be conducted to explore the effects of IOF of Arg on GH, IGF-1, and insulin. Moreover, IOF of Arg inhibits protein degradation in slow-growing chickens.

*Pax7* controls the survival of satellite cells and is the myogenic precursors that express the basic helix-loop-helic transcription factors *MyoD* and *Myf5* (44). *MyoD* is a master transcription factor for myogenic determination. The co-expression of *Pax7* and *MyoD* is correlated to the activation of satellite cells during myogenesis (45). In our study, *Pax7* and *MyoD* genes did not differ in the two groups, which is consistent with the results of Li et al. (20), who demonstrated that IOF of Arg had no effect on *Pax7* and *MyoD* in broiler chickens. The regulation of *Pax7* on *MyoD* activity may influence the muscle development (46). *Myf5* is also a member of MyoD-family in transcription factors for regulating the muscle development (47). The present study showed that *Myf5* mRNA expression was upregulated on D21 post-hatch by IOF of Arg. And, *MyoD* and *Myf5* are known to be early or committed MRFs (48). These two genes are required for the regulation of myoblast proliferation from myogenic precursor cells and for the acquisition of the myoblast phenotype (49). The loss of *MyoD* or *Myf5* functions in satellite cells led to the regeneration process failing after muscle injury (50). Kablar et al. (51) also indicated that *Myf5* expression in the limb is insufficient for myogenic development. Collectively, our results imply that satellite cells may not be activated and only *Myf5* may not be sufficient to increase the proliferation of satellite cells by IOF of Arg in slow-growing chickens.

The other two MRFs are *MyoG* and *MRF4*. *MyoG* is expressed in the first step of terminal differentiation to promote the myocyte fusion (52). Our data showed that *MyoG* mRNA expression was upregulated on DOH and D21 post-hatch, respectively. This result is consistent with previous studies, Li et al. (20) revealed that IOF of Arg increased *MyoG* gene expression in broiler chickens. Subramaniyan et al. (53) also demonstrated that *MyoG* protein was upregulated during embryogenesis by IOF of Arg. *MRF4* contributes to the later steps of myotube maturation after fusion (54). Our data showed that *MRF4* mRNA expression was increased on DOH by IOF of Arg. These results imply that both *MyoG* and *MRF4* genes may promote the terminal differentiation of satellite cells. However, it is speculated that these two genes are not mainly responsible for the enlargement of MFD because satellite cell numbers are not increased and may not form more myotubes in slow-growing chickens.

Changes in muscle mass induced by external Arg stimulation resulted from the growth of the individual muscle fibers. The *mTOR* is a major player in mediating muscle mass, which can sense intracellular changes *via* nutrient availability, and coordinate the cell growth, proliferation, differentiation, and survival (55). *Pax7* are indicator of satellite cells survival and is the myogenic precursors (4). *MyoD, Myf5, MRF4*, and *MyoG* are MRFs and play key role in muscle fiber growth and hypertrophy (56). Therefore, BMW and MFD is associated with gene expression of muscle development. The PCA results indicate that the PCA model is a good tool that clearly distinguishes the differences between the breast muscle characteristics (BMW and MFD) and gene expression of muscle development from the Arg and control group on DOH and D21 compared to D42 and D63. It is well known that Arg can improve protein synthesis and cell growth for muscle growth *via* the mTOR signaling pathway. This conclusion has been proved in poultry (18, 22). Taken together, these results suggest that IOF of Arg can play its role until starter period, and adding Arg into embryo is beneficial for the muscle development. Because of the body weight of chicken grow rapidly in the starter period, external Arg supply is necessary to meet the optimal growth of chickens. Which may be an explanation that why the previous studies focused on the effects of IOF of Arg on chickens in the starter period (18, 20, 25).

Furtherly, the positive correlations between the breast muscle characteristics (BMW and MFD) and gene expression of muscle development can be found from DOH to D63. The correlation results vary with age in these four periods, due to these genes have different expression patterns in different periods for regulating the MFD and BMW. Previous study reported that the mTOR/S6K1 pathway promoted differentiation of myogenic C2C12 cell *via* regulating *MyoD* gene, it implied that *S6K1* had ability to facilitate maturity and hypertrophy of muscle fibers (57). The deficiency of *MyoG* resulted in the decrease of body size in mice (58). Our results showed that the genes of muscle development (mTOR signaling pathway) had correlation each other in four periods, indicating that the genes of myogenesis and mTOR signaling pathway are interactive and work together for muscle development. As described in previous studies; Zhang et al. (59) found that MRFs genes were positively related to some growth traits in Tibetan chickens. Zhang et al. (9) found that the mTOR pathway was responsible for controlling the myogenic process. Rion et al. (60) also revealed that the knockdown of mTOR reduced the myogenic gene expression. In addition, a negative correlation was found on D42 and D63, respectively, which may be related to the temporal specificity of gene expression.

## Conclusion

In conclusion, IOF of 1% Arg to amnion changed the amino acid profile and improved myogenic genes (*Myf5, MyoG* and *MRF4*) expression, but did not influence muscle morphology and BMW. Thus, these findings suggest that in ovo Arg dosage (0.5 mL/egg) has no adverse effect on breast muscle development of slow-growing chickens. Further study is needed to confirm the optimal dosage of IOF of Arg for significantly improving breast muscle growth post-hatch in slow-growing chickens.

## Data availability statement

The original contributions presented in the study are included in the article/supplementary material, further inquiries can be directed to the corresponding author/s.

## Ethics statement

The animal study was reviewed and approved by the Ethics Committee on Animal Use of the Suranaree University of Technology, Nakhon Ratchasima, Thailand.

## Author contributions

PL: investigation, formal analysis, writing—original draft, and writing—review and editing. TM: investigation. AM: conceptualization, methodology, supervision, and funding acquisition. WM: conceptualization, methodology, formal analysis, writing—review editing, supervision, project administration, and funding acquisition. All authors contributed to the article and approved the submitted version.
